# Unlocking high capacitive energy-density in Sm-doped Pb(Mg_1/3_Nb_2/3_)O_3_–PbTiO_3_ thin films *via* strain and domain engineering†

**DOI:** 10.1039/d5tc00384a

**Published:** 2025-02-17

**Authors:** Zouhair Hanani, Jamal Belhadi, Nina Daneu, Urška Trstenjak, Nick A. Shepelin, Vid Bobnar, Thomas Lippert, Matjaž Spreitzer

**Affiliations:** a Advanced Materials Department, Jožef Stefan Institute Jamova cesta 39 1000 Ljubljana Slovenia zouhair.hanani@ijs.si; b Laboratory of Physics of Condensed Mater, University of Picardie Jules Verne 33 rue Saint-Leu Amiens 80039 France; c Center for Neutron and Muon Sciences, Paul Scherrer Institute Forschungsstrasse 111 5232 Villigen PSI Switzerland; d Department of Condensed Matter Physics, Jožef Stefan Institute Jamova cesta 39 1000 Ljubljana Slovenia; e Department of Chemistry and Applied Biosciences, ETH Zürich 8093 Zürich Switzerland

## Abstract

Relaxor ferroelectrics have garnered significant attention in the field of energy storage due to their exceptional properties, such as high recoverable energy density and impressive efficiency. In this work, we explore (001)-oriented and partially strained Sm-doped Pb(Mg_1/3_Nb_2/3_)O_3_–30PbTiO_3_ (Sm-PMN-30PT) thin films, prepared by pulsed laser deposition using TbScO_3_ substrates and SrRuO_3_ electrodes. We employ a comprehensive approach to evaluate the structural, compositional, and energy storage properties of Sm-PMN-30PT thin films. Our study demonstrates an ultra-high energy density of 116 J cm^−3^ and an efficiency of 73%, along with excellent thermal stability and fatigue-free energy storage properties in Sm-PMN-30PT thin films. Strain analysis of the heterostructures, performed using reciprocal space mapping (RSM) and geometric phase analysis (GPA), reveal a gradual strain relaxation across the film thickness. This results in imprinted polarization–electric field loops. Additionally, high-angle annular dark field imaging with scanning transmission electron microscopy (HAADF-STEM) depicted the existence of a strongly disordered slush-like domain structure, consisting of interconnected rhombohedral and tetragonal polymorphic nanodomains around 2–5 nm in size, and inclined “head-to-tail” polarization between neighboring domains. These results highlight the great potential of Sm-PMN-30PT films for high-capacitive energy storage devices.

## Abstract 2

Sm-doped Pb(Mg_1/3_Nb_2/3_)O_3_–30PbTiO_3_ relaxor ferroelectric thin films with slush-like structure demonstrate ultra-high energy density, excellent thermal stability and fatigue-free energy storage properties.

## Graphical abstract

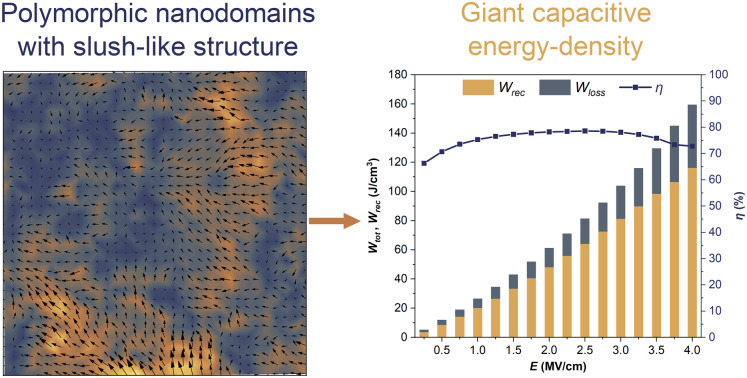

## 1. Introduction

The rapid advancement in electronics and electrical power systems necessitates enhanced electric energy storage capabilities. Electrostatic capacitors, utilizing dielectrics, are crucial for their fast charge–discharge speeds and high reliability.^1–3^ However, they suffer from lower energy densities compared to electrochemical storage systems, limiting their miniaturization and integration into compact devices.^4^ This highlights the need for advancements to improve their performance, thereby reducing the volume and cost of these devices.^5^ Dielectric capacitors store energy through the polarization under an applied electric field. The energy storage capacity (*W*_rec_) is quantified by integrating polarization values from remnant (*P*_r_) to maximum (*P*_max_) over a charge–discharge cycle, expressed as 
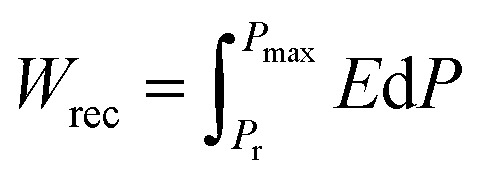
.^6,7^ A large Δ*P* = *P*_max_ − *P*_r_ value and a high breakdown field (*E*_b_) contribute to a high *W*_rec_, while a higher *P*_max_/*P*_r_ ratio, indicating reduced polarization switching hysteresis, enhances energy efficiency (
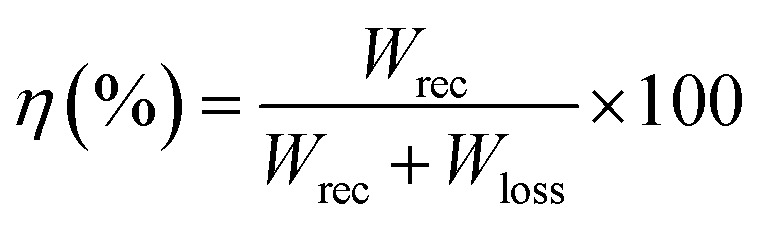
, where *W*_loss_ is the energy lost due to hysteresis).^8–10^ However, the presence of a polarization–electric field (*P*–*E*) hysteresis often compromises efficiency, restricting achievable energy density and causing significant energy loss, leading to efficiency degradation and heat dissipation issues.^9^

To address these challenges, relaxor ferroelectrics (RFEs) such as Pb(Mg_1/3_Nb_2/3_)O_3_–PbTiO_3_ (PMN–PT) and Pb_1−*x*_La_*x*_Zr_1−*y*_Ti_*y*_O_3_ (PLZT) have been developed, where that *x* represents the proportion of La substituting Pb in the perovskite structure, and *y* represents the proportion of Ti substituting Zr.^6,10–14^ These materials exhibit strong polarization but typically suffer from substantial hysteresis due to their characteristic polar domains and significant energy barriers in domain switching. To overcome this drawback, ion doping and solid solutions have transformed long-range ordered micrometer-sized ferroelectric domains into short-range ordered nanodomains.^9,15^ This enhances local heterogeneity, reduces domain switching energy barriers, decreases hysteresis, and thus improves the energy efficiency.^16–19^ Notable examples include La-doped Pb(Zr_0.52_Ti_0.48_)O_3_ films with *W*_rec_ of 68.2 J cm^−3^ and *η* of 80.4%^16^ and Sm-doped BiFeO_3_–BaTiO_3_ films with *W*_rec_ of 152 J cm^−3^ and *η* of 90%.^9^ However, this typical nanodomain strategy usually reduces the size of the polar domain entities and thus sacrifices polarization, resulting in lower energy storage densities, especially at low electric fields. To mitigate this drawback, a polymorphic nanodomain strategy based on constructing coexisting nanodomains with competitive free energies but different polarization orientations, *e.g.*, rhombohedral (*R*) and tetragonal (*T*), has been developed. The polarization anisotropy between the 〈111〉 *R* and 〈001〉 *T* orientations is greatly weakened. This leads to further flattening of polarization switching energy barriers and lower hysteresis loss while maintaining relatively high polarization.^15,20^ For instance, 0.25BiFeO_3_–0.30BaTiO_3_–0.45SrTiO_3_ thin film demonstrated an ultrahigh *W*_rec_ of 112 J cm^−3^ and high *η* of ∼80%.^15^

Recently, we reported that the introduction of Sm into Pb(Mg_1/3_Nb_2/3_)O_3_–30PbTiO_3_ thin films increased *W*_rec_ and *η* by 74 and 23%, respectively, compared to the undoped Pb(Mg_1/3_Nb_2/3_)O_3_–30PbTiO_3_.^21^ This was due to the increased local structural heterogeneity and strong local electric fields along spontaneous polarization directions facilitating nucleation of slush-like polar structure. In this study, using a nanodomain strategy combined with strain gradient engineering, we demonstrate ultra-high energy density of 116 J cm^−3^ and an efficiency of 73%, along with excellent thermal stability and fatigue-free energy storage properties under extended testing, demonstrating reliability and durability for long-term use, in Sm-PMN-30PT thin films. Such performances are attributed to the existence of a slush-like domain structure with polymorphic nanodomains, consisting of interconnected *R* and *T* with very small sizes of 2–5 nm, and the gradual strain relaxation. These improvements demonstrate the great potential of Sm-PMN-30PT films in realizing high capacitive energy storage devices.

## 2. Experimental procedures

### 2.1. Growth of the thin films using pulsed-laser deposition

2 at% Sm-doped PMN-30PT (Sm-PMN-30PT) target was prepared in-house using the columbite route. 20 mol% PbO and 10 mol% MgO excesses were used to ensure the stoichiometry of the films. (110)-TbScO_3_ single-crystal substrates (TSO, CrysTech GmBH) were annealed at high temperatures followed by chemical etching using a NaOH-deionized water solution, in order to obtain ScO_2_-terminated surface.^22^ The thin-film heterostructures were grown using pulsed laser deposition (PLD, Twente Solid State Technology, TSST) in an on-axis geometry with a target-to-substrate distance of 55 mm, using a KrF excimer laser (248 nm, LPX 300, Coherent). Before growing Sm-PMN-30PT thin films, a 35 nm-thick layer of SrRuO_3_ (SRO, Beijing Goodwill Metal Technology) serving as bottom electrode, was deposited on TSO substrates. SRO layer was deposited at 585 °C in a dynamic oxygen pressure of 0.13 mbar with a laser fluence of 2.5 J cm^−2^ and a laser repetition rate of 4 Hz, while the 250 nm-thick Sm-PMN-30PT thin films were grown at 570 °C under an oxygen partial pressure of 0.27 mbar, energy density of 2.25 J cm^−2^ and laser pulse frequency of 8 Hz. Following the growth, the samples were cooled down to room temperature at 10 °C min^−1^ in a static O_2_-pressure of 700 mbar.

### 2.2. Characterizations

The surface morphology and crystal structure of the deposited films was monitored *in situ* by reflection high-energy electron diffraction (RHEED) system (STAIB Instruments) with an accelerating voltage of 30 kV, and the patterns were analyzed using kSA 400 software (k-Space Associates). After deposition, the crystallographic properties of the thin films were characterized by X-ray diffraction (Empyrean, Malvern PANalytical) with CuKα1 radiation (*λ* = 1.5406 Å), through *θ*–2*θ* scans and reciprocal-space maps (RSMs). A double-bounce Ge (220) hybrid monochromator was used on the incident-beam side. The diffracted beam in *θ*–2*θ* scans and RSMs was captured and analyzed by a PIXcel3D detector operating in 1D mode. Furthermore, the surface morphology of the samples was examined by atomic force microscopy (AFM, Veeco Dimension 3100 SPM) using silicon probes (OTESPA-R3, Bruker), and the topographical data was thoroughly analyzed using Gwyddion AFM analysis software (https://gwyddion.net).^23^

Samples for scanning transmission electron microscopy (STEM) were prepared using Focused Ion Beam (FIB, Helios Nanolab 650) with Ga^+^ ions. STEM analyses were performed on 200 kV probe-aberration corrected microscope (JEOL ARM200 CF, Jeol Ltd.) equipped with an energy-dispersive X-ray spectrometer (EDS, Jeol Centurio 100) for the analyses of chemical composition. High-angle annular dark-field (HAADF-STEM) images were collected with a convergence angle of 24 mrad and a collection angle of 68–180 mrad, whereas bright-field images at collection angle 11–22 mrad. Strain analysis of the heterostructures was performed using geometric phase analysis (GPA).^24^ High-resolution images for the analysis of B-site displacements were acquired as stacks of 10 frames, each frame was taken with pixel time of 1.6 μs (512 × 512 px) using DigiScan Stack Acquisition Tool in Digital Micrograph Suite (Gatan, Inc.). The stacks were aligned and averaged using Stack Alignment cross-correlation procedure to obtain STEM images with improved signal to noise ratio. The positions of atomic columns in [100]_pc_ orientation (pc for pseudo-cubic) were determined using a two-dimensional Gaussian fitting procedure, and displacements for the B- and A-site columns were determined by measuring their displacements from ideal cubic positions. Image analysis and quantification were performed using atom column indexing^25^ and custom written Python scripts.

For electrical measurements, the samples were measured in parallel plate capacitor geometry using an SRO-bottom electrode and sputtered gold, using shadow masks with circular holes of 100 μm in diameter, as the top electrode. Polarization–electric field (*P*–*E*) hysteresis loops were recorded at a frequency of 100 Hz with a triangular excitation signal using a ferroelectric test system (TF Analyzer 2000E, aixACCT) equipped with a high-voltage amplifier. Fatigue measurements were performed at room temperature by completing bipolar *P*–*E* hysteresis loops at 100 Hz after applying a 100-kHz triangular waveform at 1.5 MV cm^−1^ up to ten billion cycles. The dc bias-field dependent dielectric permittivity (*κ*) and dielectric loss (tan *δ*) curves (calculated from the capacitance–voltage (*C*–*V*) characteristics) were measured at frequency of 1 kHz at a small ac signal amplitude of 50 mV by applying a dc bias field of ±0.5 MV cm^−1^ using the same ferroelectric test system.

## 3. Results and discussions

### 3.1. Structural properties

**Fig. 1:**
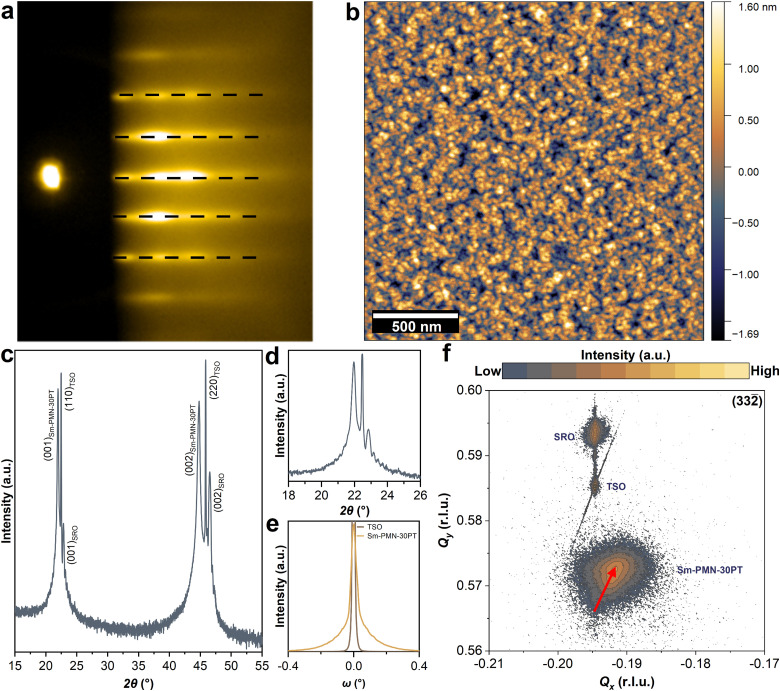
(a) RHEED pattern and (b) AFM topography image. (c) Wide-angle *θ*–2*θ* XRD pattern, (d) a zoom-in about the Sm-PMN-30PT (001)-diffraction peaks, (e) the rocking curve of Sm-PMN-30PT layer around (001)-diffraction peak, and (f) RSM around the (332̄)_pc_ reflection of the of Sm-PMN-30PT/SRO/TSO heterostructure.

The RHEED pattern of Sm-PMN-30PT thin film illustrates the formation of a pure perovskite phase during the growth as indicated by the by the dashed black lines (Fig. 1a)^12^ Besides, the RHEED pattern shows the epitaxial growth of the film in 2D and in combination with a 3D growth mode as the streaks overlapped with faint 3D transmission spots. Examination of the surface topography by AFM revealed the smoothness of the sample (root-mean-square roughness (RMS) of 0.54 nm over a scan area of 2 μm × 2 μm), as shown in Fig. 1b. To determine the crystalline quality and study the effect of the strain on the structural characteristics of Sm-PMN-30PT/SRO heterostructures, high-resolution XRD (*θ*–2*θ*, rocking curve, and RSM) was carried out. A high quality, (00l)-oriented and fully epitaxial solid solution film is obtained (Fig. 1c). The presence of Laue fringes around the main Bragg peaks is indicative of epitaxial high-quality thin-film growth, as depicted in Fig. 1d. Besides, the rocking curve obtained for Sm-PMN-30PT thin film has low full width-at-half-maximum (FWHM) values (Δ*ω* < 0.04°) that indicates a relatively low mosaicity level and confirms the high crystalline quality of the heterostructures (Fig. 1e). To further investigate the structural properties of the grown film, *i.e.*, in-plane cell parameters, strain state, and domain structures, a reciprocal space map of the asymmetric (332̄)_pc_ reflection was recorded and shown in Fig. 1f. It was found that the SRO bottom electrode is fully strained (same *Q*_*x*_ as the substrate), while the Sm-PMN-30PT film is partially relaxed with respect to the TSO substrate. The lattice parameters calculated from the RSM are *a* = 4.0132 Å and *c* = 4.0443 Å with a degree of tetragonality *c*/*a* = 1.0077. This gives rise to a compressive epitaxial lattice mismatch of −1.37%. The obtained *θ*–2*θ* scan and RSM indicate non-homogeneous lattice parameters in Sm-PMN-30PT thin film. Such variations could arise from a strain gradient due to gradual relaxation of the epitaxial strain and/or a change in the chemical composition (stoichiometry gradient) throughout the thickness of the film.^26^

### 3.2. Electrical properties

**Fig. 2:**
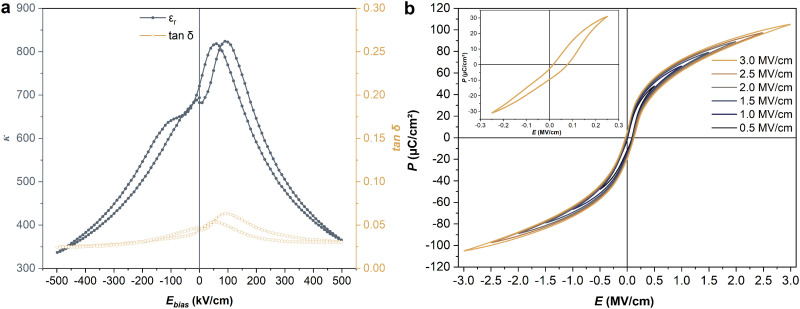
Room-temperature (a) dc-bias field dependence of the *κ* and tan *δ* (b) bipolar *P*–*E* hysteresis loops Sm-PMN-30PT thin film (the inset shows imprinted *P*–*E* hysteresis loop due to the built-in *E*_i_).

The dielectric properties *versus* bias-field of Sm-PMN-30PT thin film were recorded up to 500 kV cm^−1^ (Fig. 2a). The dc-bias field dependent dielectric permittivity (*κ*) and dielectric loss (tan *δ*) curves showed a non-linear shape with double peaks around the coercive fields suggesting the ferroelectric behavior of our samples and can be explained in terms of increased domain walls’ mobility at the coercive fields. Furthermore, the noticeable asymmetry and the positively imprinted curves could be due to various intrinsic and extrinsic factors, such as defect dipoles, trapped carriers, oxygen vacancies, strain gradients, and electrode asymmetry. For instance, Baek *et al.* attributed a large negative imprint in SRO/PMN-33PT/Pt capacitors to electrode asymmetry,^27^ while Nguyen *et al.* linked self-bias in Pb(Zr_0.52_Ti_0.48_)_0.99_Nb_0.01_O_3_ capacitors with symmetric electrodes to charge accumulation at the bottom electrode interface, driven by Nb-doping-induced strain gradients.^28^ Additionally, Kim *et al.* reported a large positive self-bias in ion-bombarded PMN–32PT capacitors, with symmetrical SRO electrodes, due to intrinsic polar defects created by high-energy ion bombardment.^10^ In this study, we believe that the significant imprint observed is not solely due to electrode asymmetry. Indeed, we attribute it to defect dipoles formed during growth to maintain charge neutrality, coupled with strain and polarization effects, as reported by Belhadi *et al.* in PMN-33PT thin films.^26^

The ferroelectricity of Sm-PMN-30PT thin film was investigated by polarization–electric field (*P*–*E*) hysteresis loops at room temperature at different electric fields, as shown in Fig. 2b. The sample presents a typical relaxor ferroelectric as evidenced by the slimmer hysteresis loop, non-saturated and high polarization (*P*_max_) and low remnant polarization (*P*_r_). At 3 MV cm^−1^, *P*_max_ and *P*_r_ reached 105.1 μC cm^−2^ and 5.8 μC cm^−2^, respectively, indicating the enhanced ferroelectricity compared to pure PMN-30PT thin film.^29^ Moreover, the *P*–*E* loops show a large electric-field shift due to the built-in internal field (*E*_i_) around 48 kV cm^−1^ (inset of Fig. 2b). The presence of large *E*_i_ in Sm-PMN-30PT thin film results in markedly large Δ*P* = *P*_max_ − *P*_r_ which is termed ferrorestorable polarization, which is highly desired in capacitive energy storage using non-linear dielectrics.^30^

### 3.3. Capacitive energy storage properties

**Fig. 3:**
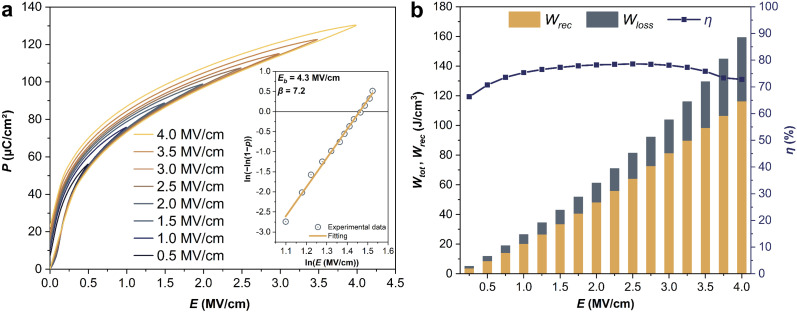
(a) Room-temperature unipolar *P*–*E* hysteresis loops (inset shows the Weibull distribution of the dielectric breakdown strength) and (b) the corresponding energy storage parameters of Sm-PMN-30PT thin film.

To identify the energy storage performance of Sm-PMN-30PT thin film, we measured unipolar *P*–*E* hysteresis loops under various electric fields at room temperature, as shown in Fig. 3a. At 4 MV cm^−1^, *P*_max_ and *P*_r_ reached 130.2 and 22.6 μC cm^−2^ corresponding to an ultra-high Δ*P* of 107.6 μC cm^−2^ and *P*_max_/*P*_r_ of 5.76. From Fig. 3b, both the total energy storage density (*W*_tot_ = *W*_rec_ + *W*_loss_) and recovered energy density (*W*_rec_) increase as the applied electric field rises (Fig. 3b). Consequently, a giant *W*_rec_ of 116.1 J cm^−3^ and *η* of 73% are simultaneously achieved at 4 MV cm^−1^. To quantitatively evaluate the trade-off between *W*_rec_ and *η*, we define a figure-of-merit 
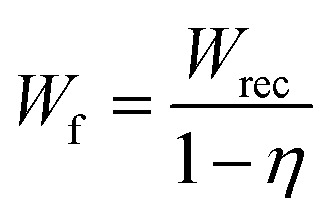
 reflecting the overall energy storage performance of the Sm-PMN-30PT thin film capacitor^31^ At 4 MV cm^−1^, *W*_f_ reached 430 J cm^−3^ which is higher than that reported for Pb-based materials^6,16,32^ (see Table S2, ESI†).

Since pulsed-power energy-storage systems are usually operated with a high applied voltage (electric field) to achieve maximum energy storage, it is important to investigate the electric-field breakdown strength *E*_b_, that is the applied electric field before dielectric breakdown occurs in the capacitors.^33^ The characteristic *E*_b_ of the Sm-PMN-30PT thin film could be analyzed using two-parameter Weibull distribution described by the formula: *X*_i_ = ln *E*_i_, *Y*_i_ = ln(−ln(1 − *p*_i_)), 
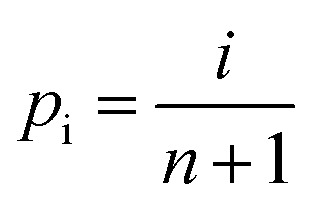
, where *E*_i_, *i*, *n* and *p*_i_ represent the tested breakdown field of each specimen, the serial number of the tested specimen, the number of the tested specimens and the cumulative probability of dielectric failure respectively. *E*_b_ is defined as the characteristic breakdown strength, which corresponds to the breakdown field at 63.2% probability of breakdown. Here, 13 breakdown field data points were collected. An *E*_b_ of 4.3 MV cm^−1^ and Weibull modulus *β* (indicating the reliability) of 7.2, were achieved (inset of Fig. 3a).

**Fig. 4:**
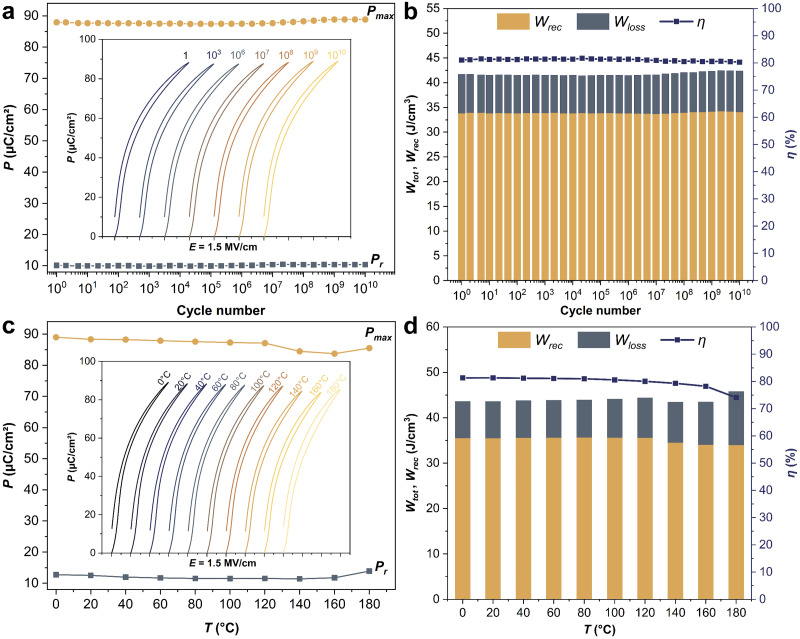
(a) Room-temperature variation of the polarization at fatigue of ten billion cycles at 1.5 MV cm^−1^ (the inset depicts the *P*–*E* hysteresis loops during cycling). (c) Temperature dependent *P*–*E* hysteresis loops between 0 and 180 °C at 1.5 MV cm^−1^ (the inset shows the *P*–*E* hysteresis loops at different temperatures). (b) and (d) The corresponding energy storage parameters of Sm-PMN-30PT thin film.

For practical applications of dielectric capacitors, the performance reliability against fatigue and stability in harsh environments are also key parameters. We tested the fatigue behaviors of the films at an electric field of 1.5 MV cm^−1^ to evaluate the cycling reliability. The ferroelectric fatigue is mainly related to the defect-pinned domain walls upon repeated switching. Besides, the repeated charging–discharging will produce joule heat, which can lead to a thermal breakdown when the generated heat exceeds the released heat. From the unipolar *P*–*E* loops after cycling (Fig. 4a), the energy storage properties were calculated and shown in Fig. 4b. The sample endured 10 billion cycles showing fatigue-free and constant energy storage properties (*W*_rec_ ∼33.8 ± 0.1 J cm^−3^, *η* ∼81.1 ± 0.4%). We also investigated the thermal stability of the energy storage performance of Sm-PMN-30PT film by recording *P*–*E* loops at the same electric field of 1.5 MV cm^−1^ from 0 to 180 °C (Fig. 4c), and plotted the temperature-dependent energy storage parameters (Fig. 4d). It can be seen that slim *P*–*E* loops do not undergo any particular change in shape along the temperature range, with little variation of *P*_max_ and *P*_r_. Correspondingly, slight fluctuations of *W*_rec_ and *η* of 4.8 and 9.7%, respectively, were observed over wide temperature range, indicating an excellent thermal stability of Sm-PMN-30PT capacitor compared to the majority of thin film capacitors reported in literature.^9,10,34–36^ Such an excellent thermal stability enables the thin film capacitors to work under severe high temperatures, for example in hybrid electric vehicles (∼140 °C).^34,37,38^

### 3.4. Origin of the enhanced capacitive energy density in Sm-PMN-30PT thin film

#### 3.4.1. Strain gradient

**Fig. 5:**
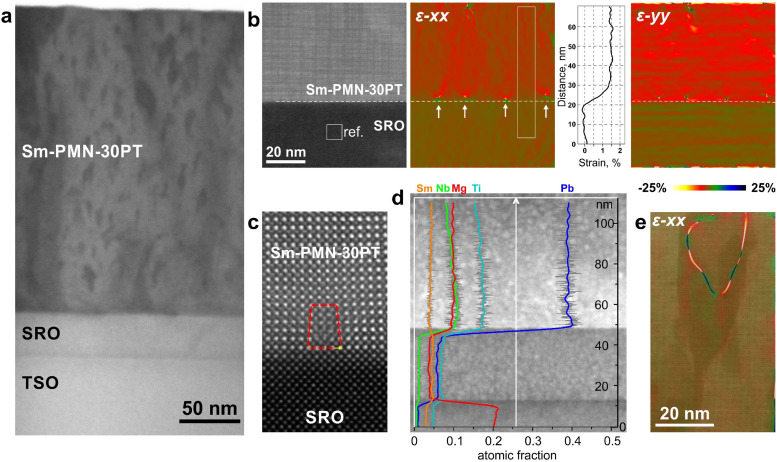
(a) BF-STEM image of the heterostructure (b) GPA analysis of the layers showing strain in the in-plane (*ε-xx*) and out-of-plane (*ε-yy*) direction. Larger variations in the out-of-plane direction coincide with the scanning direction. (c) High-resolution image of a misfit dislocation showing a missing lattice plane in the Sm-PMN-30PT film. (d) Elemental profiles constructure from quantitative EDS maps (the original data is shown in Fig. S1, ESI†) showing decreasing fraction of Nb in Sm-PMN-30PT from the contact with SRO. (e) V-shape defect with superimposed GPA analysis in the in-plane (*ε-xx*) direction.

To uncover the origin of the enhanced capacitive energy density in Sm-PMN-30PT thin films, we investigated the structural properties of the grown film, including in-plane cell parameters, strain state, and domain structures. As shown in Fig. 1f, the RSM around the (332̄)_pc_ reflection of the Sm-PMN-30PT/SRO/TSO heterostructure reveals non-homogeneous lattice parameters in the Sm-PMN-30PT layer. To further investigate this, the heterostructure was analyzed using scanning transmission electron microscopy (STEM). A low magnification bright-field STEM (BF–STEM) image of the heterostructure is shown in Fig. 5a. It depicts the Sm-PMN-30PT film with a thickness of approximately 250 nm on the TSO substrate, along with an approximately 35 nm thick SRO bottom electrode. Notably, Sm-PMN-30PT film contains darker regions resembling V-shaped defects, similar to those observed in Pb-deficient PMN–PT films on SrTiO_3_ substrates with LaNiO_3_ bottom electrodes.^39^ Furthermore, the in-plane (*ε-xx*) and out-of-plane (*ε-yy*) strains from the TSO substrate across the SRO electrode and in the first ∼60 nm of the Sm-PMN-30PT layer were analyzed using geometric phase analysis (GPA), as shown in Fig. 5b. The TSO substrate, with lattice parameters *a* and *c* of 0.396 nm, was used as the reference. Importantly, the SRO layer is fully strained to the substrate, as observed by reciprocal space mapping (RSM) (Fig. 1f), without dislocations at the interface. In the in-plane direction, the SRO lattice parameter is nearly identical (0.01% smaller) to that of the TSO substrate (0.396 nm). Consequently, the in-plane strain imposed on the SRO lattice, which otherwise has a pseudo-cubic cell of 0.393 nm, is compensated by a contraction in the out-of-plane direction by around 1.4% (to 0.390 nm).

The contact between the SRO and Sm-PMN-30PT layers is atomically sharp. However, detailed analysis reveals different mechanisms for compensating the large misfit between the smaller SRO and the larger Sm-PMN-30PT lattices. Firstly, occasional skipping of a lattice plane, *i.e.*, the formation of misfit dislocations at the SRO–Sm-PMN-30PT interface, is observed in the film (Fig. 5c). The dislocations are not periodic, as shown in the *ε-xx* image of Fig. 5b, because the misfit can also be compensated at atomic steps in the substrate. Additionally, GPA analysis shows that the in-plane lattice parameter of the Sm-PMN-30PT film in the first few tens of nanometers from the contact with the SRO varies and is larger in the areas directly above the misfit dislocations. Between the dislocations, where Sm-PMN-30PT is cube-on-cube on the underlying SRO, the in-plane lattice is more gradually relaxed, as shown in the profile calculated between the dislocations marked on the *ε-xx* image. Furthermore, relaxation of the lattice in the out-of-plane direction (*ε-yy* image in Fig. 5b) occurs immediately after the contact with the SRO. Lattice parameters of the layers in the heterostructure estimated from the GPA are given in Table S1 (ESI†). Finally, the strain in the Sm-PMN-30PT film directly after the contact with SRO is additionally compensated by small variations in the chemical composition.

Energy-dispersive X-ray spectroscopy (EDS) mapping has revealed that the fraction of the smaller Nb is higher at the contact with SRO and decreases with the distance from the contact (Fig. 5d and Fig. S1, ESI†). Although the difference is small, the preferential incorporation of Nb into the epitaxially grown Sm-PMN-30PT lattice directly after the contact with SRO contributes to the smaller lattice parameter and smaller strain. Consequently, a gradual relaxation of the Sm-PMN-30PT lattice in the in-plane direction occurs during the film growth, facilitated by the formation of V-shaped defects (Fig. 5e). In these regions, lattice discontinuities (stacking defects) in the in-plane direction are identified, whereas the lattice in the out-of-plane direction remains undisturbed.

#### 3.4.2. Nanodomain structure

**Fig. 6:**
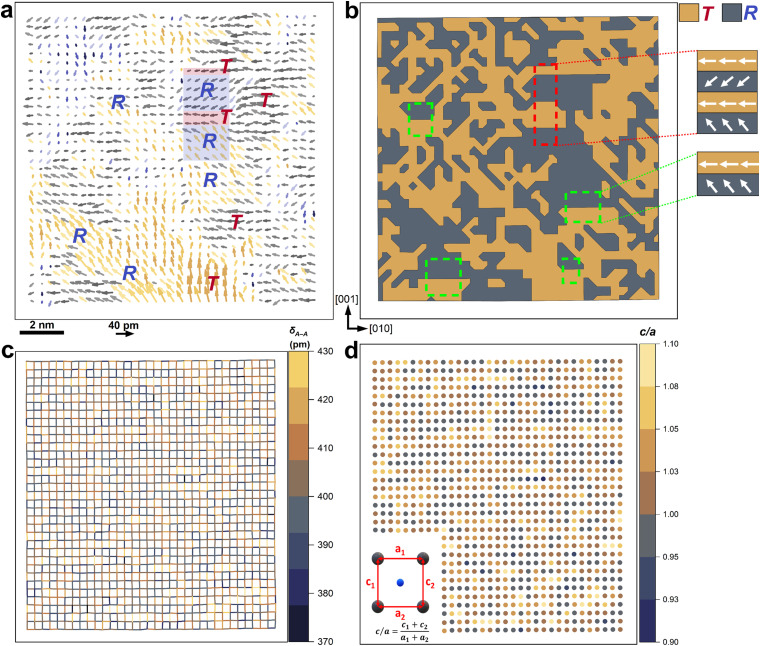
Phases and nanodomain structures of Sm-PMN-30PT thin film. (a) The extracted B-site atom displacements, where the length of arrows indicates the magnitude of the relative displacement of B-site atoms from the ideal position. (b) The classification of the *R* and *T* nanodomains generated by clustering the lattice tilts. (c) A-site positional disorder maps represented by the distance between A-site atoms. (d) Local degree of tetragonality (*c*/*a*), where the color of the circles indicates the *c*/*a* ratio for each unit cell.

To understand the local structural characteristics, *i.e.*, the phase and nanodomain structure, we further analyzed the Sm-PMN-30PT thin film using high-angle annular dark field imaging with scanning transmission electron microscopy (HAADF-STEM). The original HAADF-STEM image is shown in Fig. S2a (ESI†), where the perovskite A and B sites are visualized as bigger (brighter) and smaller (darker) spots, respectively. For this purpose, we analyzed the B-site atom off-center displacements (represented by arrows) within their A-site cages (Fig. 6a). Notably, non-isolated nanodomain structures with diameters between approximately 2 nm and 5 nm are observed, forming a unique slush-like polar structure with a multi-domain state (Fig. 6b).^40,41^ This configuration is typical of PMN–PT materials.^41^ Additionally, the magnitude map of B-site atom displacements (*δ*_B_), presented in Fig. S2b (ESI†), indicates that the displacements are not uniform but rather clustered into nanoregions of similar displacement direction and magnitude, with a maximum *δ*_B_ of 54 pm. Using a polar plot, we estimated the average B-site atom displacements (*δ*_B_) of 15.4 ± 8.1 pm between the [01̄0]_pc_ and [01̄1]_pc_ directions (Fig. S3, ESI†).

We classified the nanodomain structure into two groups using the experimentally observed lattice displacement, by clustering neighboring sites within ±22.5° of four diagonal directions, with each color representing a preferred orientation lattice displacement (Fig. 6b). Here gold color represents preferred lattice displacement along the [010]_pc_, [001]_pc_, [01̄0]_pc_, and [001̄]_pc_, while the grey color denotes preferred lattice displacement along the [011]_pc_, [01̄1]_pc_, [01̄1̄]_pc_, and [011̄]_pc_ directions. We observed tetragonal and rhombohedral-like (projected in [01̄1]_pc_ and [01̄1̄]_pc_ directions) domains, with “head-to-tail” polarization configuration, as illustrated in the insets of Fig. 6b. It is worth mentioning that the coexistence of different ferroelectric structures with varying symmetries provides considerable scope for polarization rotation *via* a reduction of polarization anisotropy, which significantly impacts the domain structure.

Additionally, we analyzed the A-sublattice positional disorder in Sm-PMN-30PT thin film using STEM by calculating the distances between A-site atoms (*δ*_A–A_) on a per-unit cell basis, as shown in Fig. 6c. The effective lattice parameter is approximately 403.3 ± 10.1 pm, suggesting a significantly large local fluctuation. This is reflected by the large number of brighter segments in Fig. 6c. The incorporation of Sm^3+^ induces A-site distortion due to their smaller ionic radius compared to Pb^2+^, which increases lattice heterogeneity and disrupts long-range ferroelectric order, facilitating a more dynamic response to external fields.^21^ These strong local A-site distortions are hypothesized to play a key role in the short-range ordering and thus the relaxor behavior of the RFE compositions.^42^ Moreover, we assessed the degree of tetragonality 
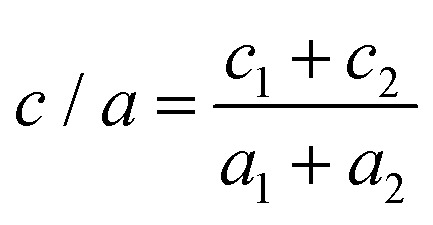
 at the A-site positions (Fig. 6d) and observed high and fluctuated *c*/*a* values with an average of 1.0124 ± 0.0289, suggesting an averagely tetragonal structure in Sm-PMN-30PT thin film. These findings are similar to those reported by Li *et al.* in Sm-PMN-30PT single crystal, stating that the introduction of Sm^3+^ dopants on the A sites of the PMN-30PT crystal created disruptions in the long-range ferroelectric domains and move the PMN-30PT composition towards the morphotropic phase boundary (MPB).^43^ These observations indicate that the introduction of Sm^3+^ not only induces local A-site distortions but also enhances the relaxor behavior and structural complexity of the Sm-PMN-30PT thin film.

In a single-domain film, a unipolar state at saturation, where 180° domains are absent, can be achieved using an external electric field. However, after discharging, only partial relaxation of the polarization occurs, leading to poor recovered energy density.^44^ In contrast, Sm-PMN-30PT thin films with engineered polydomain structures and inclined “head-to-tail” polarization between neighboring domains, relative to the electric field direction, require a much larger electric field to become fully oriented. This results in a significantly higher energy density. Furthermore, the suppressed remanent polarization in these films leads to excellent energy efficiency. Additionally, the structural and compositional disorders in Sm-PMN-30PT thin films are critical for optimizing their energy storage properties and overall performance in capacitive applications.

The ultrahigh energy storage properties in Sm-PMN-30PT thin films cannot be attributed to a single mechanism but is rather a complex combination of different contributions originating from the multiple effects of samarium doping on the long- and short-range structure of PMN–PT. The Sm-doping enhances local structural heterogeneity and MPB characteristics, including polarization vector instability, reduced domain size, low-angle domain walls, and metastable phase coexistence.^45^ This enables polarization rotation by reducing anisotropy, improving domain structure and functional properties. Besides, Li *et al.* attributed these effects to softening caused by Sm^3+^ ions replacing Pb^2+^ ions, and the reduction of oxygen vacancy concentration from donor doping may also enhance polar nanoregion mobility, facilitating polarization rotation.^46^ Additionally, the high density of low-angle domain walls ensures robust thermal stability of energy density in Sm-PMN-30PT thin films.^21^

## 4. Conclusion

In summary, Sm-PMN-30PT thin film was successfully prepared on TSO substrate using an SRO oxide electrode buffer layer by pulsed laser deposition. A high recoverable energy density of 116 J cm^−3^ and an energy efficiency of 73%, excellent cycling reliability (up to 10^10^ charge–discharge cycles), and good temperature stability (from 0 to 180 °C) were achieved. Using an array of structural and composition characterizations, we revealed that the origin of these enhanced properties arises from the presence of the polymorphic domain structure and the gradual strain relaxation. This later resulted in ferrorestorable polarization due to the built-in internal electric field and enhanced recoverable energy density. Furthermore, the Sm-doping created higher local structural heterogeneity and enhanced the MPB characteristics, where several phenomena coexisted, including the instability of the polarization vector against rotation, reduction of domain size, conditions favoring the stabilization of phases with lower symmetry, and the metastable coexistence of multiple phases. The coexistence of *R* and *T* ferroelectric structures with inclined “head-to-tail” polarization between neighboring domains provided considerable scope for polarization rotation *via* a reduction of polarization anisotropy. These findings positioned Sm-PMN-30PT relaxor ferroelectric as a promising candidate for capacitive energy storage applications.

## Data availability

All data supporting the findings of this study are available within the paper and its ESI.†

## Conflicts of interest

There are no conflicts to declare.

## Supplementary Material

TC-013-D5TC00384A-s001
