# Supplementary material for: Unlocking high capacitive energy-density in Sm-doped Pb(Mg1/3Nb2/3)O3–PbTiO3 thin films via strain and domain engineering
Source: J Mater Chem C Mater. 2025 Feb 17;13(14):7140–9. doi: 10.1039/d5tc00384a (PMC11870079; doi:10.1039/d5tc00384a)
Supplement: TC-013-D5TC00384A-s001 [file TC-013-D5TC00384A-s001.pdf]

Supplementary Information (SI) for Journal of Materials Chemistry C.
This journal is © The Royal Society of Chemistry 2025

Supporting Information

Unlocking high capacitive energy-density in Sm-doped $Pb(Mg_{1/3}Nb_{2/3})O_3$–$PbTiO_3$ thin films *via* strain and domain engineering

Zouhair Hanani[1,*], Jamal Belhadi[2], Nina Daneu[1], Urška Trstenjak[1], Nick A. Shepelin[3], Vid Bobnar[4], Thomas Lippert[3,5], and Matjaž Spreitzer[1]

[1] Advanced Materials Department, Jožef Stefan Institute, Jamova cesta 39, 1000, Ljubljana, Slovenia.

[2] Laboratory of Physics of Condensed Mater, University of Picardie Jules Verne, 33 rue Saint-Leu, Amiens 80039, France.

[3] Center for Neutron and Muon Sciences, Paul Scherrer Institute, Forschungsstrasse 111, 5232 Villigen PSI, Switzerland.

[4] Department of Condensed Matter Physics, Jožef Stefan Institute, Jamova cesta 39, 1000 Ljubljana, Slovenia.

[5] Department of Chemistry and Applied Biosciences, ETH Zürich, 8093 Zürich, Switzerland.

*Corresponding author. Email: zouhair.hanani@ijs.si

1. **Chemical compositions of Sm-PMN-30PT thin film**

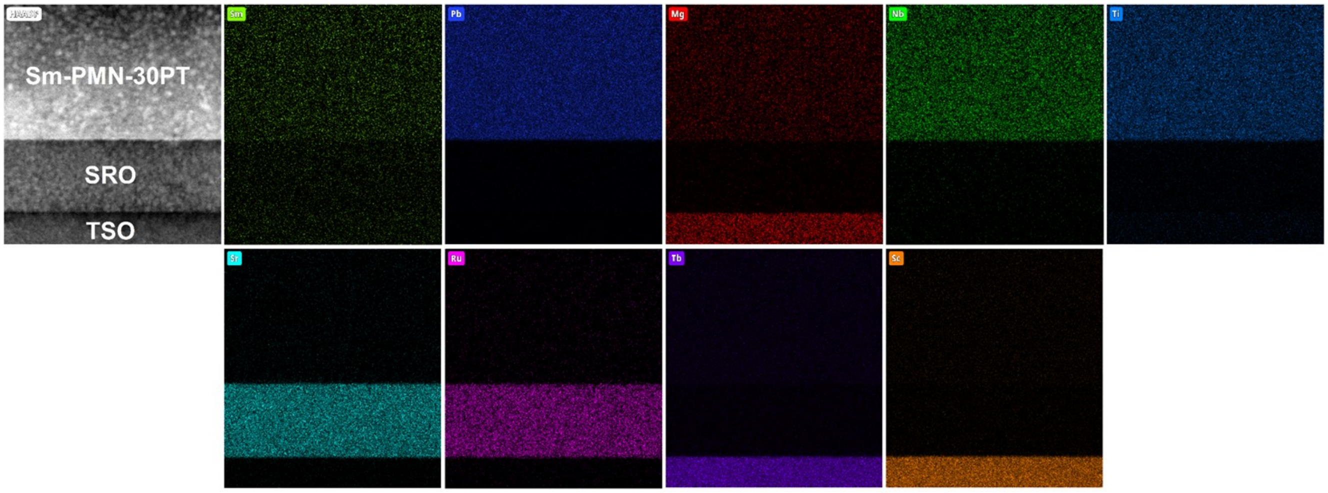

Fig. S1. Chemical compositions of Sm-PMN-30PT thin film. Bright-field TEM image showing the heterostructure Sm-PMN-30PT/SRO/TSO and EDS elemental mappings of Sm, Pb, Mg, Nb, Ti, Sr, Ru, Tb, and Sc elements.

2. Lattice strain and lattice parameters of Sm-PMN-30PT thin film

Table S1: Evaluation of lattice strain (in percentage) and lattice parameters based on GPA analysis of HAADF-STEM images shown in Figure S1.

		a		***c***	
		Strain (%)	*Dimension (nm)*	*Strain (%)*	*Dimension (nm)*
**Fig. S1a**	*TSO substrate*	Reference	0.396	Reference	0.396
	SRO electrode	−0.01	0.396	−1.5	0.390
**Fig. S1b**	*SRO electrode*	Reference	0.396	Reference	0.390
	Sm-PMN-30PT layer	+1.5 (gradual)	0.402	+3.4	0.403

3. Phases and nanodomain structures of Sm-PMN-30PT thin film

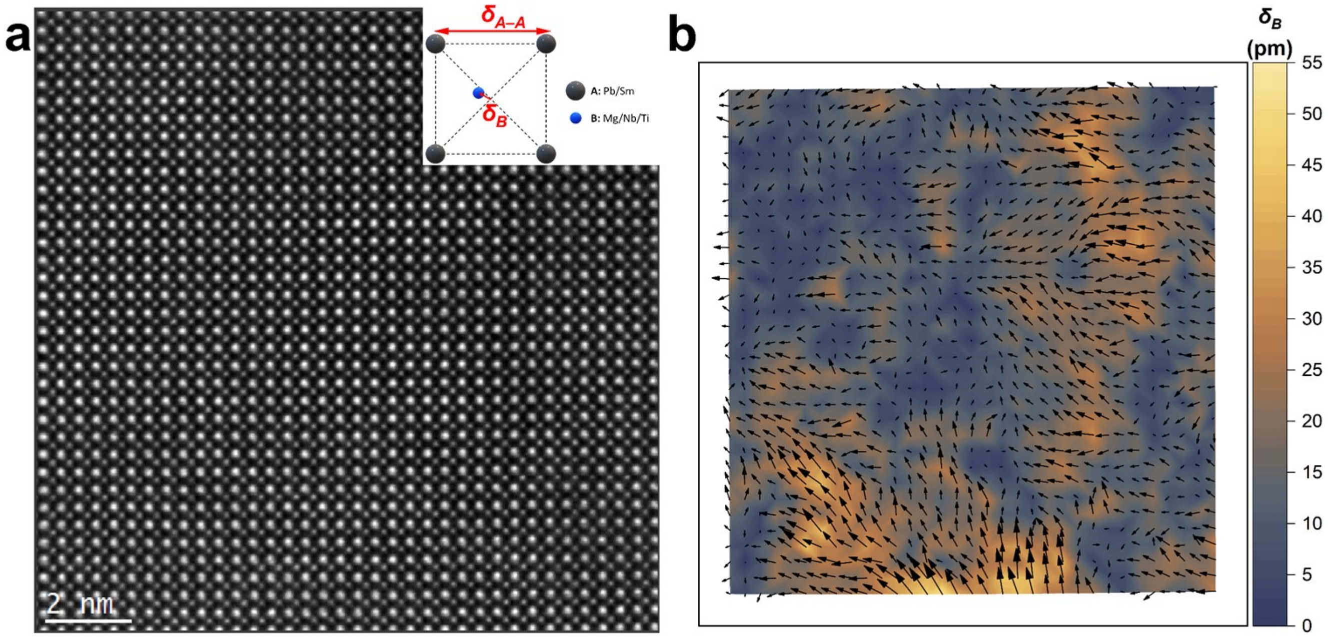

Fig. S2. Phases and nanodomain structures of Sm-PMN-30PT thin film. (a) Atomic resolution HAADF-STEM images of a 35 × 35 unit-cell area viewed along the $[100]_{pc}$ zone axis. The larger spots correspond to the A-site columns and smaller spots to the B-site columns. The inset shows the B-site displacements (δ_B) from the center of four neighboring A-site atoms of the perovskite unit cell ($\delta_{A\text{–}A}$ is the distance between A-site atoms). (b) Vector map of B-site displacements away from the center of the A-site perovskite sublattice. Arrows mark the direction and magnitude (arrow length) of the displacements, and the contours additionally mark their magnitude.

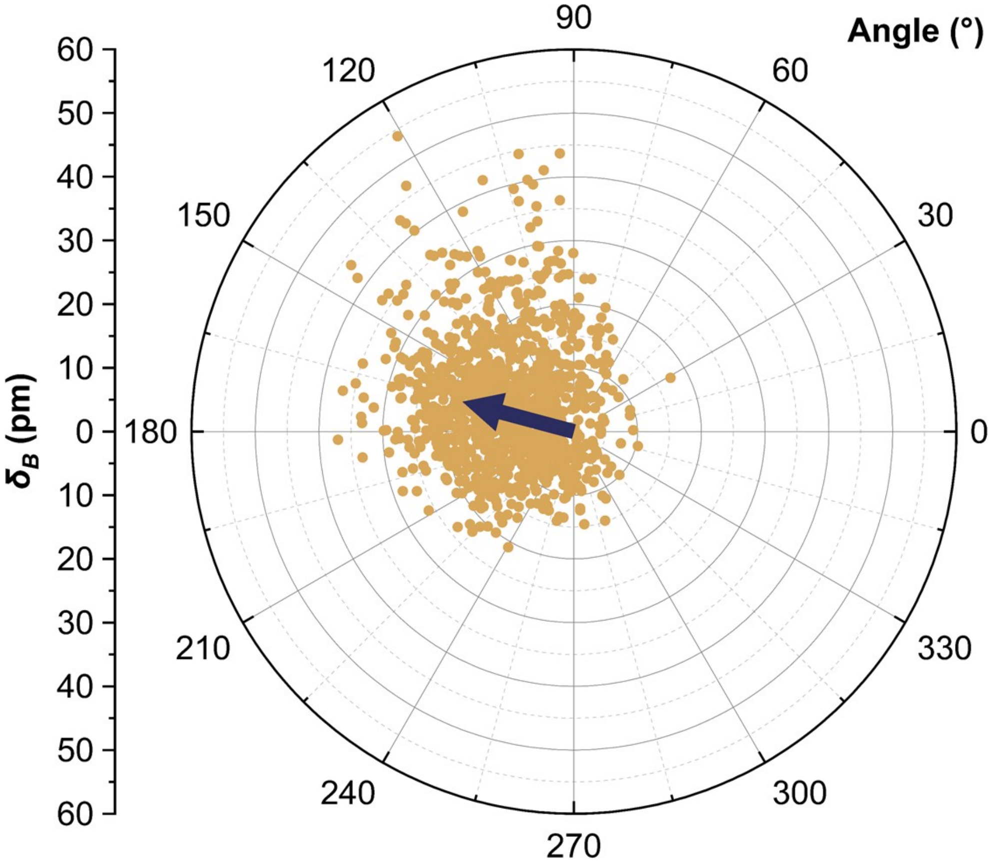

Fig. S3. Polar plot of the B-site atom displacements. The arrow indicates the average magnitude and angle of the displacement.

Table S1. Comparison of the energy storage parameters (W_{rec}, η, E_{max} and W_{rec}/E_{max}) of Sm-PMN-30PT and other relaxor ferroelectric thin films reported in literature.

Dielectric films	E_{max} (MV cm^{-1})	W_{rec} (J cm^{-3})	η (%)	W_{rec}/E_{max} (J MV^{-1} cm^{-2})	Q_F (kJ cm^{-3})	Ref.
Sm-PMN-30PT	4	116.1	73	30.0	430	This work
30 mol% Sm-doped $0.3BiFeO_3$-$0.7BaTiO_3$	5.2	152	77	29.2	660.9	[1]
($0.7Na_{0.5}Bi_{0.5}TiO_3$-$0.3SrTiO_3$)/($0.6SrTiO_3$-$0.4Na_{0.5}Bi_{0.5}TiO_3$)	2.61	60	51	23.0	122.4	[2]
$0.25BiFeO_3$-$0.30BaTiO_3$-$0.45SrTiO_3$	4.9	112	80	22.9	560.0	[3]
$0.68Pb(Mg_{1/3}Nb_{2/3})O_3$-$0.32PbTiO_3$	5.92	133.3	75	22.5	533.2	[4]
($0.4BiFeO_3$-$0.6SrTiO_3$)/$Ba_{0.5}Sr_{0.5}TiO_3$	4.76	98	80	20.6	490.0	[5]
$0.30BiFeO_3$-$0.35BaTiO_3$-$0.35SrTiO_3$	4	79	78	19.8	359.1	[6]
La-doped $0.9Na_{0.5}Bi_{0.5}TiO_3$-$0.1BiFeO_3$	2.7	52.4	60.3	19.4	132.0	[7]
$0.9Na_{0.5}Bi_{0.5}TiO_3$-$0.1BiFeO_3$	2	38.5	52	19.3	80.2	[8]
$Pb_{0.9}La_{0.1}(Zr_{0.52}Ti_{0.48})O_3$	3.6	68.2	80.4	18.9	348.0	[9]
$Na_{0.5}Bi_{0.5}TiO_3$	1.25	23.3	61.6	18.6	60.7	[10]
$0.4BiFeO_3$-$0.6SrTiO_3$	3.85	70.3	70	18.3	234.3	[11]
$Pb_{0.9}La_{0.1}(Zr_{0.52}Ti_{0.48})O_3/Pb(Zr_{0.52}Ti_{0.48})_{0.99}Nb_{0.01}O_3$	2.45	43.5	84.1	17.8	273.6	[12]
$0.65Pb(Mg_{1/3}Nb_{2/3})O_3$-$0.35PbTiO_3$	2	35	70	17.5	116.7	[13]
$0.88Ba_{0.55}Sr_{0.45}TiO_3$-$0.12BiMg_{2/3}Nb_{1/3}O_3$	5	86	73	17.2	318.5	[14]
$0.25BiFeO_3$-$0.75SrTiO_3$	4.46	70	68	15.7	218.8	[11]
Mn-doped $Pb_{0.97}La_{0.02}(Zr_{0.905}Sn_{0.015}Ti_{0.08})O_3$	2	31.2	58	15.6	74.3	[15]
0.5 mol% Mn-doped $0.4BiFeO_3$-$0.6SrTiO_3$	3.6	51	64	14.2	141.7	[16]
$Pb_{0.9}La_{0.1}Zr_{0.52}Ti_{0.48}O_3$	3	40.9	80.2	13.6	206.6	[17]
$0.6PbTiO_3$-$0.4Bi(Mg_{0.5}Zr_{0.5})O_3$	2.6	32.3	51.4	12.4	66.5	[18]
$Ba_{0.7}Ca_{0.3}TiO_3/BaZr_{0.2}Ti_{0.8}O_3$	4.5	52.4	72.3	11.6	189.2	[19]
$BaZr_{0.35}Ti_{0.65}O_3$	8.7	100.8	78	11.6	458.2	[20]
Sm-doped $BaZr_{0.2}Ti_{0.8}O_3$	3.68	40.42	85	11.0	270.0	[21]
0.01 mol% Mn-doped $0.55Na_{0.5}Bi_{0.5}TiO_3$-$0.45Sr_{0.2}Bi_{0.7}TiO_3$	2.86	30.5	65	10.7	87.1	[22]

$BaZr_{0.2}Ti_{0.8}O_3$	3	30.4	81.7	10.1	166.1	[23]

References

[1] Pan H, Lan S, Xu S, Zhang Q, Yao H, Liu Y, et al. Ultrahigh energy storage in superparaelectric relaxor ferroelectrics. Science (80-) 2021;374:100–4. https://doi.org/10.1126/science.abi7687.

[2] Zhang Y, Li W, Xu S, Wang Z, Zhao Y, Li J, et al. Interlayer coupling to enhance the energy storage performance of Na0.5Bi0.5TiO3-SrTiO3 multilayer films with the electric field amplifying effect. J Mater Chem A 2018;6:24550–9. https://doi.org/10.1039/c8ta09396b.

[3] Pan H, Li F, Liu Y, Zhang Q, Wang M, Lan S, et al. Ultrahigh–energy density lead-free dielectric films via polymorphic nanodomain design. Science (80-) 2019;365:578–82. https://doi.org/10.1126/science.aaw8109.

[4] Kim J, Saremi S, Acharya M, Velarde G, Parsonnet E, Donahue P, et al. Ultrahigh capacitive energy density in ion-bombarded relaxor ferroelectric films. Science (80-) 2020;369:81–4. https://doi.org/10.1126/science.abb0631.

[5] Lv P, Qian J, Yang C, Wang Y, Wang W, Huang S, et al. 4-inch Ternary BiFeO3-BaTiO3-SrTiO3Thin Film Capacitor with High Energy Storage Performance. ACS Energy Lett 2021;6:3873–81. https://doi.org/10.1021/acsenergylett.1c02017.

[6] Pan H, Feng N, Xu X, Li W, Zhang Q, Lan S, et al. Enhanced electric resistivity and dielectric energy storage by vacancy defect complex. Energy Storage Mater 2021;42:836–44. https://doi.org/10.1016/j.ensm.2021.08.027.

[7] Wang F, Chen J, Tang Z, Guo F, Zhao S. High energy storage properties for the lead-free NBT-0.1BFO-0.068La relaxor ferroelectric film. J Alloys Compd 2021;854:157306. https://doi.org/10.1016/j.jallcom.2020.157306.

[8] Wang F, Zhu C, Zhao S. High energy storage density of NBT-0.10BFO solid solution films. Ceram Int 2021;47:8653–8. https://doi.org/10.1016/j.ceramint.2020.11.235.

[9] Nguyen MD, Nguyen CTQ, Vu HN, Rijnders G. Experimental evidence of breakdown strength and its effect on energy-storage performance in normal and relaxor ferroelectric films. Curr Appl Phys 2019;19:1040–5. https://doi.org/10.1016/j.cap.2019.06.005.

[10] Wang F, Zhu C, Zhao S. Good energy storage properties of Na0.5Bi0.5TiO3 thin films. J Alloys Compd 2021;869:159366. https://doi.org/10.1016/j.jallcom.2021.159366.

[11] Ma J, Pan H, Ma J, Zhang Q, Liu X, Guan B, et al. Giant energy density and high efficiency achieved in bismuth ferrite-based film capacitors via domain engineering. Nat Commun 2018;9:1813. https://doi.org/10.1038/s41467-018-04189-6.

[12] Nguyen MD, Houwman EP, Do MT, Rijnders G. Relaxor-ferroelectric thin film heterostructure with large imprint for high energy-storage performance at low operating voltage. Energy Storage Mater 2020;25:193–201. https://doi.org/10.1016/j.ensm.2019.10.015.

[13] Park CK, Lee SH, Lim JH, Ryu J, Choi DH, Jeong DY. Nano-size grains and high density of 65PMN-35PT thick film for high energy storage capacitor. Ceram Int 2018;44:20111–4. https://doi.org/10.1016/j.ceramint.2018.07.303.

[14] Fan Y, Zhou Z, Chen Y, Huang W, Dong X. A novel lead-free and high-performance barium strontium titanate-based thin film capacitor with ultrahigh energy storage density and giant power density. J Mater Chem C 2019;8:50–7. https://doi.org/10.1039/c9tc04036f.

[15] Peng B, Tang S, Lu L, Zhang Q, Huang H, Bai G, et al. Low-temperature-poling awakened high dielectric breakdown strength and outstanding improvement of discharge energy density of (Pb,La)(Zr,Sn,Ti)O3 relaxor thin film. Nano Energy 2020;77:105132. https://doi.org/10.1016/j.nanoen.2020.105132.

[16] Pan H, Zeng Y, Shen Y, Lin YH, Ma J, Li L, et al. BiFeO3-SrTiO3 thin film as a new lead-free relaxor-ferroelectric capacitor with ultrahigh energy storage performance. J Mater Chem A 2017;5:5920–6. https://doi.org/10.1039/c7ta00665a.

[17] Nguyen CTQ, Vu HN, Nguyen MD. High-performance energy storage and breakdown strength of low-temperature laser-deposited relaxor PLZT thin films on flexible Ti-foils. J Alloys Compd 2019;802:422–9. https://doi.org/10.1016/j.jallcom.2019.06.205.

[18] Wang C, Sun N, Hao X. Dielectric property and energy-storage performance of (1–x)PbTiO3–xBi(Mg0.5Zr0.5)O3 relaxor ferroelectric thin films. J Mater Sci Mater Electron 2020;31:2063–72. https://doi.org/10.1007/s10854-019-02727-6.

[19] Sun Z, Ma C, Liu M, Cui J, Lu L, Lu J, et al. Ultrahigh Energy Storage Performance of Lead-Free Oxide Multilayer Film Capacitors via Interface Engineering. Adv Mater 2017;29. https://doi.org/10.1002/adma.201604427.

[20] Liang Z, Ma C, Dai Y, Du X, Liu M. Effect of mosaicity on energy storage performance of epitaxial BaZr0.35Ti0.65O3 films. Appl Phys Lett 2021;118. https://doi.org/10.1063/5.0044987.

[21] Sun Z, Tian X, Shang L, Hao X, Wang G, Shi Y, et al. Modifying energy storage performances of new lead-free system ferroelectric capacitors through interfacial stress. Appl Surf Sci 2021;559:149992. https://doi.org/10.1016/j.apsusc.2021.149992.

[22] Wang J, Qiu G, Qian H, Liu Y, Luo J, Lyu Y. Optimized energy-storage performance in Mn-doped Na0.5Bi0.5TiO3-Sr0.7Bi0.2TiO3 lead-free dielectric thin films. Appl Surf Sci 2022;571:151274. https://doi.org/10.1016/j.apsusc.2021.151274.

[23] Sun Z, Ma C, Wang X, Liu M, Lu L, Wu M, et al. Large Energy Density, Excellent Thermal Stability, and High Cycling Endurance of Lead-Free BaZr0.2Ti0.8O3 Film Capacitors. ACS Appl Mater Interfaces 2017;9:17096–101. https://doi.org/10.1021/acsami.7b03263.
